# Stage-Dependent Impact of RIPK1 Inhibition on Atherogenesis: Dual Effects on Inflammation and Foam Cell Dynamics

**DOI:** 10.3389/fcvm.2021.715337

**Published:** 2021-10-25

**Authors:** Yuze Zhang, Huihui Li, Yonghu Huang, Hong Chen, Haojie Rao, Guoli Yang, Qing Wan, Zekun Peng, John Bertin, Brad Geddes, Michael Reilly, Jean-Luc Tran, Miao Wang

**Affiliations:** ^1^State Key Laboratory of Cardiovascular Disease and Clinical Pharmacology Center, Fuwai Hospital, National Center for Cardiovascular Diseases, Chinese Academy of Medical Sciences and Peking Union Medical College, Beijing, China; ^2^Innate Immunity Research Unit, GlaxoSmithKline, Collegeville, PA, United States

**Keywords:** atherosclerosis, RIPK1, foam cells, macrophages, inflammation, reverse cholesterol transport, cell death

## Abstract

**Objective:** Atherosclerosis is an arterial occlusive disease with hypercholesterolemia and hypertension as common risk factors. Advanced-stage stenotic plaque, which features inflammation and necrotic core formation, is the major reason for clinical intervention. Receptor interacting serine/threonine-protein kinase 1 (RIPK1) mediates inflammation and cell death and is expressed in atherosclerotic lesions. The role of RIPK1 in advanced-stage atherosclerosis is unknown.

**Approach and Results:** To investigate the effect of RIPK1 inhibition in advanced atherosclerotic plaque formation, we used *ApoE*^*SA*/*SA*^ mice, which exhibit hypercholesterolemia, and develop angiotensin-II mediated hypertension upon administration of doxycycline in drinking water. These mice readily develop severe atherosclerosis, including that in coronary arteries. Eight-week-old *ApoE*^*SA*/*SA*^ mice were randomized to orally receive a highly selective RIPK1 inhibitor (RIPK1i, GSK547) mixed with a western diet, or control diet. RIPK1i administration reduced atherosclerotic plaque lesion area at 2 weeks of treatment, consistent with suppressed inflammation (MCP-1, IL-1β, TNF-α) and reduced monocyte infiltration. However, administration of RIPK1i unexpectedly exacerbated atherosclerosis at 4 weeks of treatment, concomitant with increased macrophages and lipid deposition in the plaques. Incubation of isolated macrophages with oxidized LDL resulted in foam cell formation *in vitro*. RIPK1i treatment promoted such foam cell formation while suppressing the death of these cells. Accordingly, RIPK1i upregulated the expression of lipid metabolism-related genes (*Cd36, Ppara, Lxr*α, *Lxrb, Srebp1c*) in macrophage foam cells with ABCA1/ABCG1 unaltered. Furthermore, RIPK1i treatment inhibited ApoA1 synthesis in the liver and reduced plasma HDL levels.

**Conclusion:** RIPK1 modulates the development of atherosclerosis in a stage-dependent manner, implicating both pro-atherosclerotic (monocyte infiltration and inflammation) and anti-atherosclerotic effects (suppressing foam cell accumulation and promoting ApoA1 synthesis). It is critical to identify an optimal therapeutic duration for potential clinical use of RIPK1 inhibitor in atherosclerosis or other related disease indications.

## Introduction

Atherosclerotic cardiovascular disease is a leading cause of morbidity and mortality worldwide (1). Development of atherosclerosis involves lipid metabolism disorder and inflammation and is substantially modified by risk factors. Monocytes are initially recruited to the vessel wall in response to local stimulation by modified lipids and differentiate into macrophages, which uptake oxidated lipids and mediate cholesterol efflux to liver via a process called reverse cholesterol transport (RCT) (2). The recruited macrophages phagocytize oxidized low-density-lipoproteins (LDL) via CD36 (3), diminishing lipid accumulation in the artery. As atherosclerosis proceeds, with more oxidized LDL deposit, an imbalance between the uptake and degradative metabolism of oxidized LDL drives macrophages to transform into lipid-rich foam cells. In advanced stage atherosclerosis, macrophages/foam cells cannot effectively process the engulfed cholesterol, undergo cell death and release lipid contents, forming necrotic core. With lipid necrotic core expansion and increasing inflammation, plaque may become vulnerable, with its rupture causing acute atherothrombotic manifestations, such as myocardial infarction (4, 5). High-density-lipoprotein (HDL) exerts athero-protection by transporting excess cholesterol from macrophages to liver (RCT) (6). Apolipoprotein A1 (ApoA1), mainly synthesized in the liver and the small intestine, is a major structural protein of HDL that accepts cholesterol exported from macrophages during RCT. Expression of lipoproteins is regulated by transcription factors, such as peroxisome proliferator-activated receptors (PPAR) (7, 8). Hypercholesterolemia and hypertension are two common risk factors for atherosclerosis (9), and their combination exerts a strong synergistic effect on inducing advanced atherosclerotic plaques.

Receptor interacting serine/threonine-protein kinase 1 (RIPK1), a member of serine/threonine kinases family, mediates TNFα-induced programmed cell death, namely, apoptosis and necroptosis (10). Activation of RIPK1-dependent apoptosis, triggered by a secondary cytosolic Complex IIa which includes RIPK1, FADD, and caspase-8 (11), has been implicated in some inflammatory diseases (12). RIPK1 could mediate an alternative form of regulated necrotic cell death called necroptosis via sequential phosphorylation of RIPK3. In this process, mixed lineage kinase domain-like protein (MLKL) is recruited and phosphorylated to facilitate membrane rupture and cell lysis (11). RIPK1 also plays an essential role in the activation of IkB kinase/mitogen-activated protein kinase pathway under TNFα induction. It influences transcription of cellular FLICE-like inhibitory protein and release of nuclear factor κ-light-chain-enhancer of activated B cells (NF-κB) (13). Due to its crucial role in cell death and inflammation, RIPK1 has implicated as a potential therapeutic target for cardiovascular diseases. RIPK1 is expressed in human and mouse atherosclerosis lesions (14), highly in macrophages (15). RIPK1 Inhibition is protective in myocardial infarction (16) and early atherosclerosis (17) in mice. By contrast, in a recent study, loss of MLKL, a downstream molecule of RIPK1/RIPK3 mediated necroptosis pathway, increased macrophage lipid accumulation in atherosclerosis (18). The role of RIPK1 in the development of advanced atherosclerosis, which features both inflammation and cell death, remains unclear.

Here, we report that GSK547 (RIPK1i), a mono-selective kinase inhibitor that robustly targets RIPK1 *in vivo* (19) plays a dual role in lipid metabolism and inflammation, conferring a stage-dependent impact on atherosclerosis.

## Materials and Methods

### Mice

Eight-weeks-old male and female *ApoE*^*SA*/*SA*^ mice on C57BL/6J background, which are deficient in *Apoe* gene and with SR-BI knockdown and inducible angiotensin II expression (by doxycycline administration) were used in the present study (20). For atherosclerosis study, *ApoE*^*SA*/*SA*^ mice were fed a Western diet (TD 88137; Harlan Teklad) which contains GSK547 powder [GlaxoSmithKline; 10 mg/kg/day (0.083 g/kg food)]. Doxycycline (Dox) (1 mg/ml, protected from the light, Sangon Biotech, Shanghai, China) water was prepared freshly and replaced every other day to induce hypertension for 2 or 4 weeks. All animal experiments were approved by the Institutional Animal Care and Use Committee, Fuwai Hospital, National Center for Cardiovascular Diseases, China.

### Histologic Analysis

After being sacrificed, fresh mice hearts were collected after perfused via the left ventricle with 5 ml PBS, and embedded in OCT. Aortic sinus serial sections were cut through the aorta at the origin of the aortic valve leaflets for 4 cross-sections (8 μm), at 80 μm intervals. In this study, cross sections with three valves present were considered as representative images of the aortic sinus. Aortic sinus atherosclerotic lesions were stained with Oil Red O (Sigma, USA), and the average of Oil Red O (ORO)-positive area at different levels in aortic sinus were used to represent each animal. Coronary artery ORO-positive area was assessed in five transverse sections at 200 μm intervals. All ORO-positive area in the five cardiac sections along the coronary arteries was calculated and the average value was used to represent each animal. Weigert staining (Leagene, China) was used to show elastic fiber. Next, the stained slides were scanned with a Pannoramic SCAN (3DHistech) histological scanner. Quantification analysis of the lesions were measured manually using Image-Pro Plus 6.0 software (Media Cybernetics, MD). For immunofluorescent staining, frozen sections were firstly placed at room temperature for 20 min, then fixed in 95% alcohol for 10 min and transferred to phosphate buffer to remove OCT. Tissue sections were blocked with goat serum containing 0.3% of Triton X-100 for 60 min and then incubated with primary antibodies overnight at 4°C, followed by incubation with specific secondary antibody for 1 h at 37°C. Coverslips were mounted with a medium containing DAPI (Zsbio, China) to stain nuclei. Images were taken by an Axiovert 200M microscope (Zeiss, Germany) or by Pannoramic SCAN (3DHistech, Hungary). The antibodies used for immunofluorescent staining were as follows: F4/80 (1:50, sc52664, Santa Cruz, Dallas, TX, USA), RIPK3 (1:100, sc-374639, Santa Cruz, Dallas, TX, USA), α-SMA (1:200, ab5694, Abcam, Cambridge, UK).

### Acute TNF/zVAD Shock Model

Acute TNF + zVAD shock model was used to examine activity of RIPK1 inhibitor *in vivo* as previously described (19). Mice were pretreated with 0.01, 0.1, 1, and 10 mg/kg GSK547 administered orally, then challenged with injection with TNF/zVAD-fmk (16.7 mg/kg). Body temperature was determined as readout for GSK547 efficacy in protecting from shock induced by TNF/zVAD.

### Pharmacokinetic Experiment

Mice were administered GSK547 orally at 0.01, 0.1, 1.0, and 10 mg/kg, then blood drug levels at serial time points were measured. The average drug level from 0.5 h post-dose was used to calculate the predicted RIPK1 inhibition, and actual body temperature change was calculated as percent change corrected to the TNF/zVAD group.

### Chronic Food Base Pharmacokinetic and TNF-Induced Shock Experiments

RIPK1 inhibitor GSK547 PK/PD study was conducted to assess drug levels in mice fed the diet for 1 week at 9.6 and 96 mg/kg/day. Mice were challenged on day 8 with TNF/zVAD at the same dose mentioned above.

### Evaluation of Plaque Vulnerability

Plaque vulnerability in aortic sinus cross-sections from both groups of mice was evaluated by H&E staining and immunohistochemistry. Necrotic core was quantified by acellular area as described (21). Smooth muscle cells content in plaque was quantified by α-SMA positive area.

### Plasma Lipid Measurement

Sodium citrate anti-clotted blood samples were collected from inferior vena cava after mice were anesthetized. Plasma was isolated from blood after centrifugation (8,000 rpm, 4°C, 5 min) and stored in −80°C freezer until analysis. Plasma concentration of total cholesterol (TC) and HDL-C were measured using commercial kits (BioSino, China) and analyzed by UniCel DxC 800 Synchron (Backman, USA).

### Cytokines Analysis

Plasma cytokine secretion was evaluated by Meso QuickPlex SQ120 (Meso Scale Discovery, Rockville, MD, USA).

### Cell Study

Peritoneal macrophages isolated from male *ApoE*^*SA*/*SA*^ mice or male C57BL/6J mice, which were pretreated with RIPK1i (50 ng/ml) or DMSO for 3 h; Then, the isolated macrophages were stimulated with oxidized LDL (50 μg/ml; YB-002, Yiyuan Biotechnologies, China) for 24 h. Macrophages were stained with Oil Red O, Hoechst33342 (Thermo, USA) or TUNEL (Roche, Switzerland), and were photographed by photomicroscope (Leica, Germany). For each well, five consecutive microscopic pictures were taken randomly. The counts of ORO or TUNEL positive cell was quantified using the Image J software.

### RNA Isolation and qPCR

For RNA isolation, peritoneal macrophages or liver isolated from mice were homogenized in 1 ml TRIzol (Invitrogen, USA). The solution was then mixed with 200 μl chloroform, and centrifuged (12,000 rpm; 15 min; 4°C). The aqueous phase was removed, mixed with same volume of isopropanol and centrifuged (12,000 rpm; 10 min; 4°C). After washing with 75% ethanol, the concentration of RNA was determined by ultraviolet spectrophotometer. A total of 500 ng RNA was used for reverse transcription using PrimeScript™RT Master Mix (Takara, Japan). qPCR reactions were performed in triplicate with SYBR master mix (Yeasen, China). The primers used in this study are listed as follows:

**Table:** 

**Gene**	**Forward primer (5′-3′)**	**Reverse primer (5′-3′)**
*Cd36*	TGGCAAAGAACAGCAGCAAA	AGGAGCACAACTTGAACAAATGA
*Apoa1*	GGCACGTATGGCAGCAAGAT	CCCAGAAGTCCCGAGTCAAT
*Apom*	CCTGGGCCTGTGGTACTTTA	CCATGTTTCCTTTCCCTTCA
*Ppara*	TGTCTGTCGGGATGTCACACA	AAGCGTCTTCTCGGCCATAC
*Lxra*	GGAGTGTCGACTTCGCAAATG	TCAAGCGGATCTGTTCTTCTGA
*Lxrb*	TCCGACCAGCCCAAAGTCA	GCTTGGCAAAGTCCACAATCTC
*Srebp1c*	GGAGCCATGGATTGCACATT	GCTTCCAGAGAGGCCAG
*Gapdh*	GGTTGTCTCCTGCGACTTCA	GGTGGTCCAGGGTTTCTTACTC

### Western Blot Analyses

For western blot analyses, liver from male *ApoE*^*SA*/*SA*^ mice were lysed in RIPA buffer containing protease inhibitors (P0013, Beyotime Biotechnology, China). After full cracked, homogenate was centrifuged at 10,000–14,000 g for 3–5 min, and the supernatant was collected into another EP tube. Supernatant was mixed with loading buffer and denatured by heating. Proteins were subjected to 10% SDS-PAGE and then transferred to PVDF membranes (Thermo, USA). Immunoblotting was performed for ApoA1 (1:1,000, ab20453, Abcam, UK) and β-actin (1:5,000, ab8226, Abcam, UK). Horseradish peroxidase-conjugated secondary antibodies (1:10,000, Yeasen, China,) and ECL luminous liquid (Thermo, USA) were used to detect proteins on a FluorChem System (ProteinSimple, USA).

### Flow Cytometry

Macrophages were incubated with AF647-ABCA1 (1:500, MCA2681A647, BioRad, Hercules, CA, USA) or PE/Cy7-ABCG1 (1:2,000, NB400-132PECY7, Novus Biologicals, Centennial, CO, USA). Samples were acquired on a FACS Aria II (BD Biosciences, USA) flow cytometer. Analysis of the flow cytometry data was performed using FlowJo (BD Biosciences, USA).

### Blood Pressure Measurement

Mouse systolic blood pressure was measured by a tail-cuff method (IITC Life Science, Woodland Hills, CA, USA) as reported previously (22). Before the atherosclerosis study, mice were trained 3 days for acclimatization of tail-cuff method. From the beginning of induction of hypertension, mouse systolic blood pressure measurement was measured at a fixed time every day.

### Myocardial Ischemia-Reperfusion

Wild-type C57BL6/J mice were subjected to 30-min ligation of the coronary artery and 24-hr reperfusion, as we previously described (23). GSK547 (50 mg/kg) was administered by intraperitoneal injection immediately after the coronary ligation and 12 h after the coronary ligation. Cardiac infarct size was quantified by TTC staining at the end of reperfusion.

### Quantification of Aneurysms

Abdominal aorta aneurysms (AAA) were analyzed after aortae were isolated from the mice and cleaned of perivascular tissues, as previously described (24).

### Statistical Analysis

Statistical analysis was performed using GraphPad Prism 8 software (GraphPad Software, San Diego, USA). Comparisons of the two groups were made using unpaired *t*-test or non-parametric Mann-Whitney test as appropriate. Comparison among >2 groups was performed by a one-way ANOVA followed by the *post-hoc* analysis with an unpaired *t*-test or non-parametric Mann-Whitney test to evaluate statistical significance between the two groups. Statistical significance was defined as *P* < 0.05. In text and graphs, data were presented as the mean ± standard error.

## Results

### GSK547 Pharmacokinetics and Its Potent Inhibition of RIPK1 in Mice

**Figure 1:**
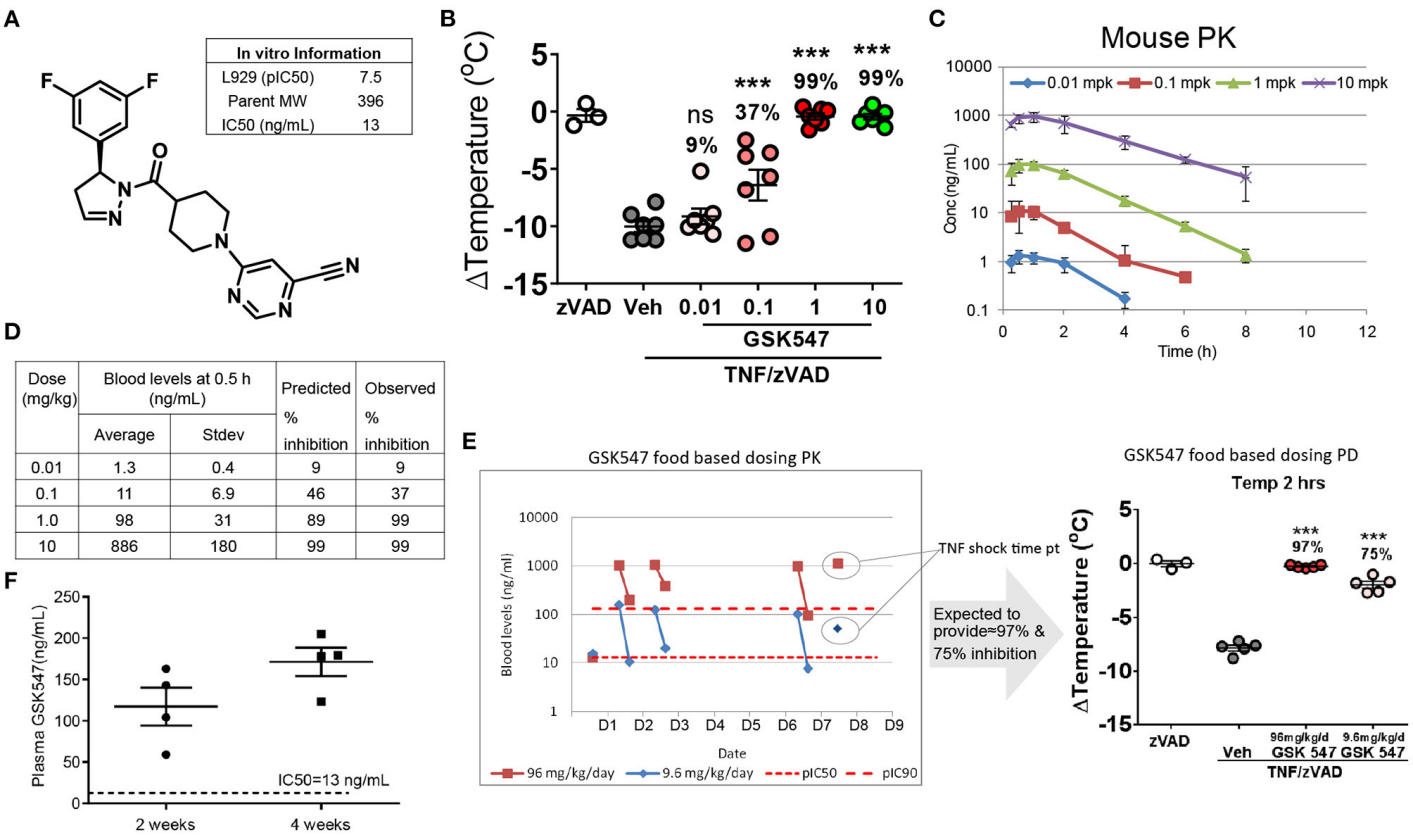
Pharmacokinetics of GSK547 and its potent inhibition of RIPK1 in mice. **(A)** Chemical structure of GSK547 and its reported *in vitro* IC50 (19). **(B)** Pharmacodynamics dose-response in an acute TNF/zVAD shock model on body temperature at 0.01, 0.1, 1, and 10 mg/kg GSK547 administered orally. mpk = mg/kg. *n* = 3–7 mice per group. Mice were administered GSK547 orally at 0.01, 0.1, 1.0, and 10 mg/kg, then blood drug levels at serial time points were shown **(C,D)**. The average drug level from 0.5 h post-dose was used to calculate the predicted RIPK1 inhibition, and actual body temperature change was calculated as percent change corrected to the TNF/zVAD group. **(C)** Pharmacokinetics of GSK547 at 0.01, 0.1, 1, and 10 mg/kg orally. *n* = 5 mice per group. **(D)** Correlation of PK drug level to the RIPK1inhibtion. The drug concentration is correlated with the acute *in vivo* PK data on **(D)** at 0.1 mpk (11 ng/ml), 1 mpk (98 ng/ml), 10 mpk (886 ng/ml). The drug dose of 0.1 mpk is equivalent to the IC50 of the *in vitro* data. The drug concentration increased dose-dependently, which correlated well to the predicted and observed inhibition as indicated in the table **(B,D)**. At 1.0 and 10 mpk doses, the GSK547 inhibited RIPK1 activation at 99%. **(E)** Administration of GSK547 in food base PK study achieved a steady-state concentration. GSK547 PK (**E**, left)/PD study (**E**, right) was conducted and blood was sampled at peak and trough in mice fed the diet for 1 week at 9.6 and 96 mg/kg/day. Mice were challenged on day 8 with TNF/zVAD (16.7 mg/kg). n=3-5 mice per group. **(F)** Plasma levels of GSK547 in *ApoE*^*SA*/*SA*^ mice that were administered with a western diet (21% fat and 0.2% cholesterol) mixed with GSK547 at a dose of 10 mg/kg/day, and with Doxycycline (Dox)-containing water to induce hypertension. Sample was collected at ~10 a.m. of the day after 2- and 4-week dosing. *n* = 4 mice per group. All the data represented as mean ± SEM; ^*^^*^^*^P < 0.001. Statistical analysis: Ordinary one-way ANOVA test.

GSK547 is a mono-selective kinase inhibitor (Figure 1A) that robustly targets RIPK1, with an *in vitro* IC50 of 13 ng/ml (19). GSK547 dose-dependently suppressed acute TNF/zVAD shock in mice (Figure 1B). Consistent with the acute *in vivo* efficacy on body temperature change protection, the pharmacokinetics of GSK547 in mice displayed a well-correlated dose-dependent pharmacokinetics/pharmacodynamics (PK/PD) relationship (Figure 1C). The RIPK1 inhibition prediction was calculated based on the *in vitro* potency of pIC50 at 7.5 and the *in vivo* PK drug level (Figure 1D). The GSK547 would inhibit 99 % of RIPK1 at 10 mg/kg, which was validated in the actual observation from the experiment. To further validate the *in vivo* utility of GSK547 for the chronic dosing, the PK study was conducted in a food-based study, at doses of 9.6 and 96 mg/kg. The blood samples were collected at ~8:00 a.m. for peak and 3:00 p.m. for trough on the animal eating cycle on days 1, 2, and 6 (Figure 1E), and mice were challenged with TNF/zVAD shock on day 8. As shown, the GSK547 drug level consistently achieved the concentration needed to inhibit RIPK1. Therefore, RIPK1i GSK547 is a desirable molecule with great PK/PD for the *in vivo* chronic study.

### Differential Impacts of RIPK1 Inhibition on Early and Late Atherogenesis in Mice

**Figure 2:**
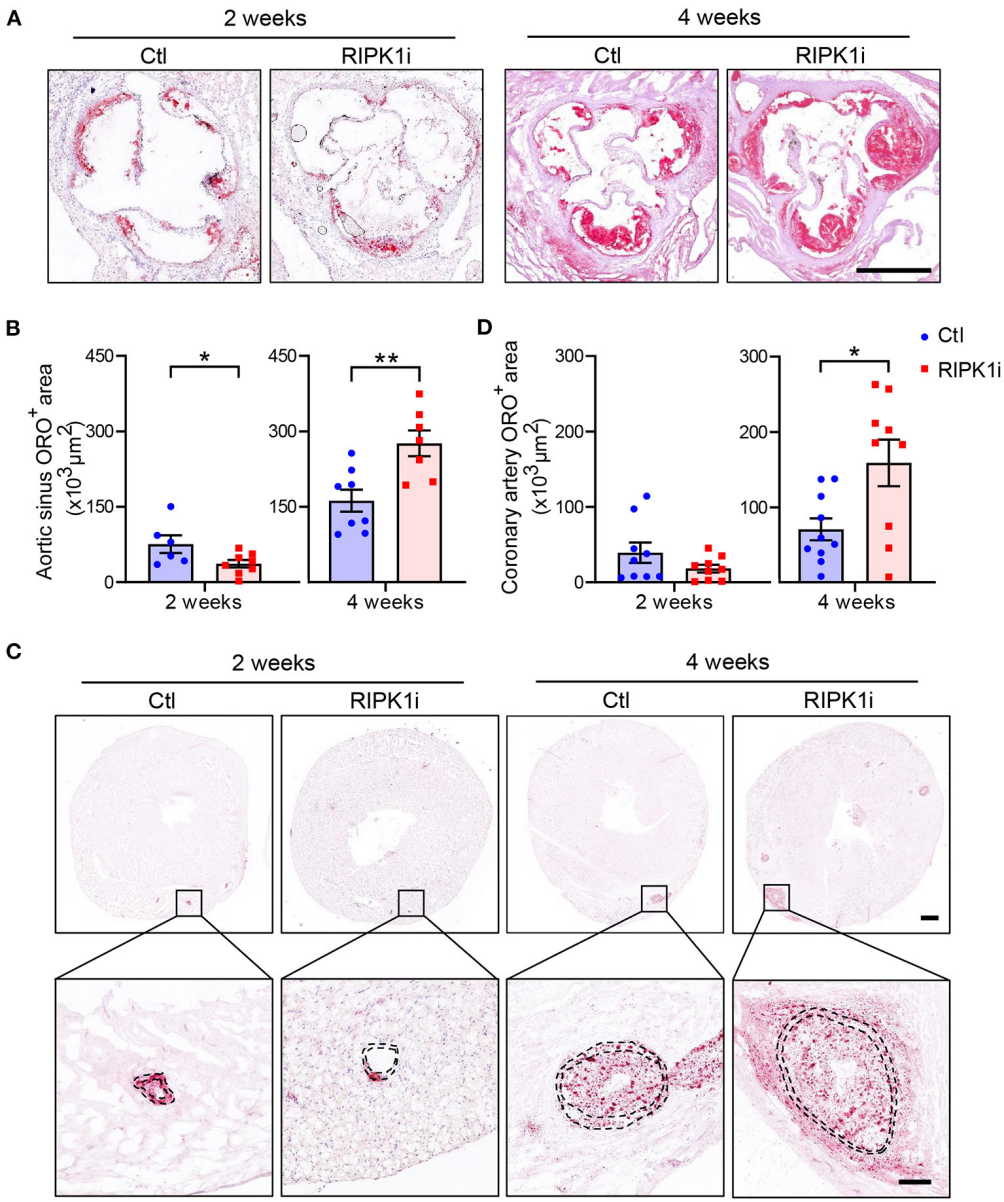
Differential impacts of RIPK1 inhibition on early and late atherogenesis in mice. Male *ApoE*^*SA*/*SA*^ mice were fed a western diet contained RIPK1 inhibitor GSK547 (RIPK1i, 10 mg/kg) or not (Ctl) for 2 or 4 weeks with Dox-containing water to induce hypertension. Representative histological analysis of cross-sections from the aortic sinus stained with Oil Red O **(A)** and quantification of aortic sinus Oil Red O-positive area of male mice **(B)**. Scale bar = 500 μm. Representative histological analysis of heart cross-sections stained with Oil Red O **(C)** and quantification of coronary artery **(D)** Oil Red O-positive area of male mice. Panel **C**, scale bar = 500 μm, top; scale bar = 100 μm, bottom. Dashed lines showed vessel media. Blue symbols = Ctl group, pink symbols = RIPK1i group. *n* = 6–10 mice per group. All the data represented as mean ± SEM; ^*^P < 0.05, ^*^^*^P < 0.01. Statistical analysis: unpaired student's t test.

To examine the effect of RIPK1 inhibition on the development of advanced atherogenesis, we used a mouse model (*ApoE*^*SA*/*SA*^) that exhibits both hypercholesterolemia and hypertension upon induction and develop advanced atherosclerotic lesions including those in coronary arteries (20). Eight-week-old male *ApoE*^*SA*/*SA*^ mice were administered with a western diet (21% fat and 0.2% cholesterol) mixed with or without GSK547 at a dose of 10 mg/kg/day, and with doxycycline (Dox)-containing water to induce hypertension. The plasma levels of GSK547, as determined at 2 and 4 weeks of treatment (Figure 1F), were 117.3 and 171.3 ng/ml, respectively, which is >9 folds of IC50 during administration. In male mice, RIPK1i reduced plaque lesion areas in aortic sinus at 2 weeks of the atherosclerosis induction. However, prolonged treatment of RIPK1i exacerbated atherosclerotic lesion formation at 4 weeks of the atherosclerosis induction (Figures 2A,B). Likewise, coronary artery ORO-positive area was ameliorated at early stage, but enhanced at late stage (Figures 2C,D). Similar to male mice, 8-week-old female *ApoE*^*SA*/*SA*^ mice treated with RIPK1i developed more atherosclerosis lesions in late stage (Supplementary Figures S1A,B). We used Weigert staining to show elastic fibers of coronary arteries (Supplementary Figure S1C) and no difference was observed between RIPK1i and control group in coronary artery media thickness (68,273 ± 6,510, 66,840 ± 23,550; *t*-test with Welch's correction, *p* = 0.8811, *n* = 7,8). RIPK1i treatment did not alter blood pressure (Supplementary Figure S2A). No difference was observed in abdominal aortic aneurysm formation (Supplementary Figures S2B,C).

### RIPK1i Treatment Decreased Plasma HDL and Suppressed Liver ApoA1 Expression

**Figure 3:**
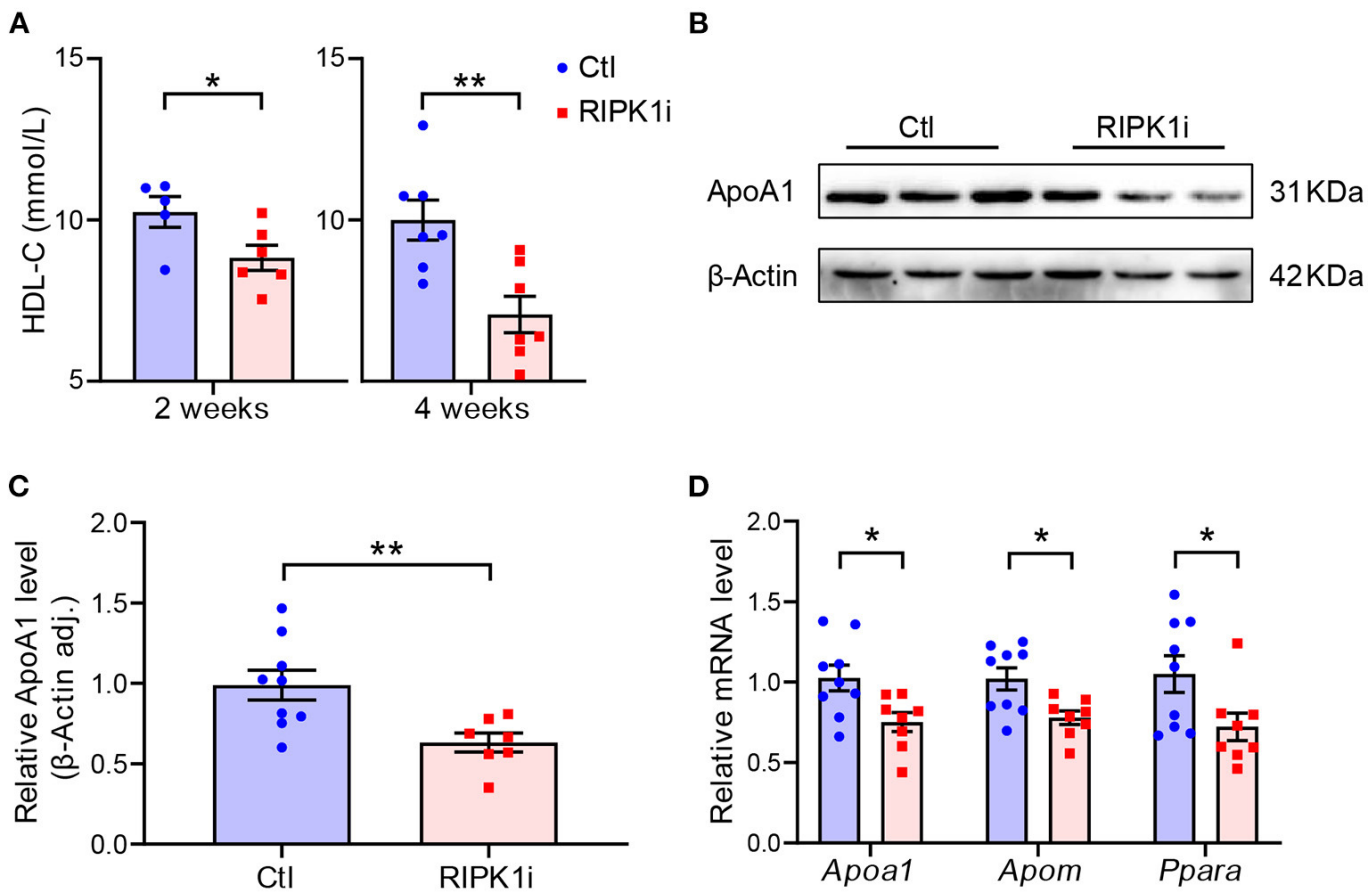
RIPK1i treatment decreased plasma HDL and suppressed liver ApoA1 expression level. **(A)** Plasma HDL-C level in male *ApoE*^*SA*/*SA*^ mice fed with WD and Dox for 2 and 4 weeks. *n* = 5–8 mice per group. **(B)** Representative Western blot images of ApoA1 from liver of male *ApoE*^*SA*/*SA*^ mice fed with WD and Dox for 2 weeks. **(C)** Quantification of liver ApoA1 protein level (*n* = 9.7). **(D)** qRT-PCR analysis of *Apoa1, Apom, Ppara* in liver of male *ApoE*^*SA*/*SA*^ mice fed with WD and Dox for 2 weeks (*n* = 9.8). All of the data represented as mean ± SEM; ^*^P < 0.05, ^*^^*^P < 0.01. Statistical analysis: unpaired student's *t*-test.

To understand the impact of RIPK1 inhibition on atherogenesis, we first examined plasma lipids level in the mice. HDL-C level was progressively lowered in RIPK1i groups compared with control groups (Figure 3A), whereas total cholesterol level showed no significant difference compared to control groups (Supplementary Figure S3). ApoA1, the major protein component of HDL, is synthesized mainly in the liver, and its expression was thus detected. RIPK1i treatment markedly decreased ApoA1 protein expression (Figures 3B,C) and mRNA expression level (Figure 3D). Meanwhile, liver expression level of apolipoprotein M (ApoM), an HDL-associated plasma protein exhibiting various anti-atherosclerotic functions such as anti-oxidation and pro-cholesterol efflux, was also decreased by RIPK1i. Consistently, expression of *Ppara*, a transcription factor of ApoA1, was also reduced (Figure 3D). Hence, RIPK1 inhibition caused lipid metabolism dysfunction, which might impair the RCT process, facilitating atherogenesis.

### Long-Term RIPK1i Treatment Promoted Macrophage Accumulation and Foam Cell Formation

**Figure 4:**
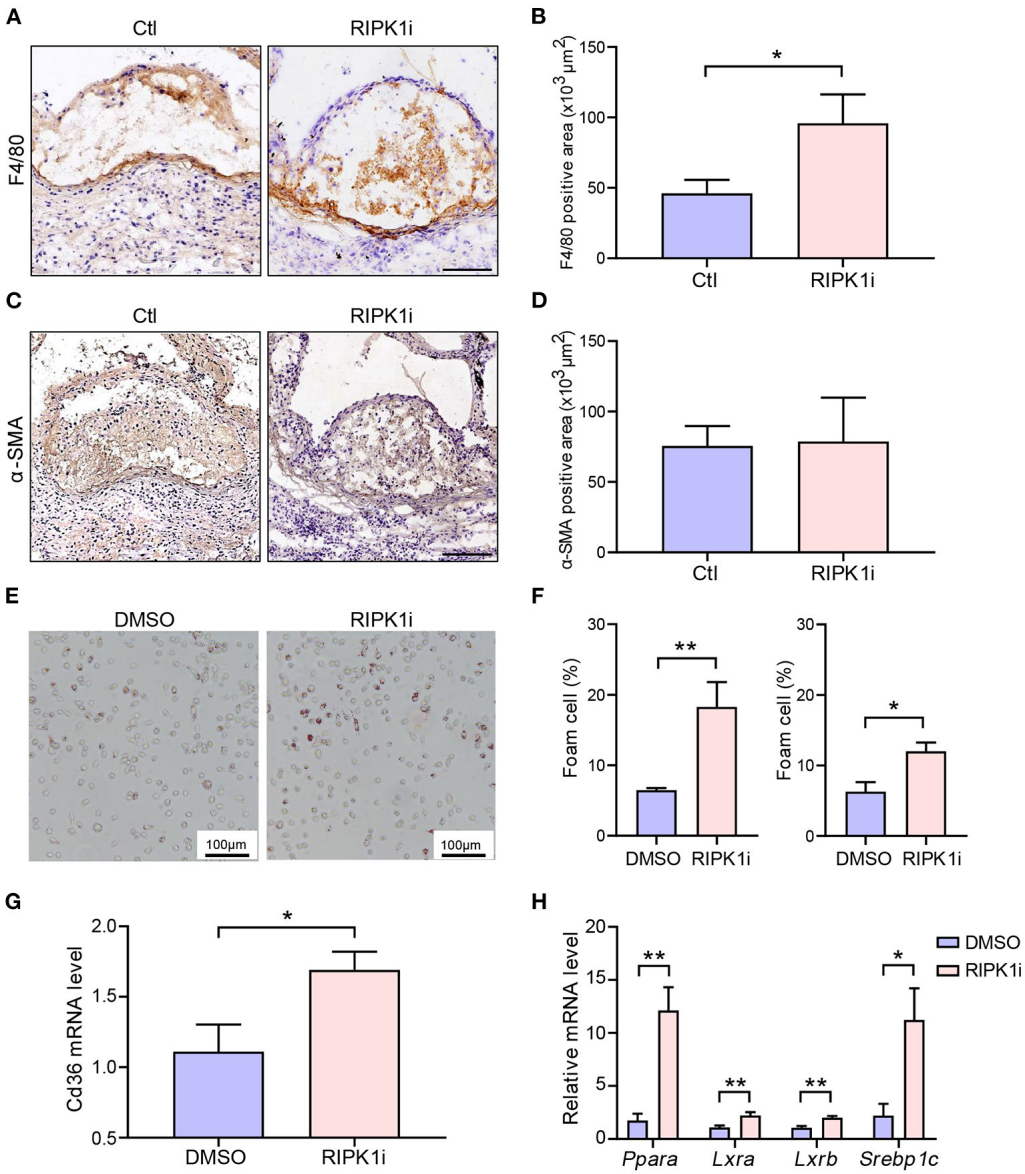
RIPK1i accelerated macrophage accumulation and foam cell formation. **(A–D)** Histological analysis of cross-sections from aortic sinus of *ApoE*^*SA*/*SA*^ mice receiving RIPK1i for 4 weeks. *n* = 5 mice per group. **(A)** Representative IHC images of F4/80 in aortic sinus plaque. Scale bar = 100 μm. **(B)** Quantification of macrophages (F4/80 positive) are represented. **(C)** Representative IHC images of α-SMA in aortic sinus plaque. Scale bar = 100 μm. **(D)** Quantification of smooth muscle cells (α-SMA positive) are represented. **(E–H**) Peritoneal macrophages isolated from male *ApoE*^*SA*/*SA*^ mice or male C57BL/6J mice were pretreated with RIPK1i (50 ng/ml) or DMSO for 3 h, then stimulated with oxidized LDL (50 μg/ml) for 24 h. **(E)** Representative images of Oil Red O stained foam cells for male *ApoE*^*SA*/*SA*^ mice. **(F)** Percentage of foam cells in ox-LDL induced peritoneal macrophages for male *ApoE*^*SA*/*SA*^ mice (left, *n* = 5) and male wild-type mice (right, *n* = 3). **(G)** Relative *Cd36* mRNA level in ox-LDL induced peritoneal macrophages for male *ApoE*^*SA*/*SA*^ mice (*n* = 6). **(H)** Lipid metabolism-related genes expression levels in ox-LDL induced peritoneal macrophages for male *ApoE*^*SA*/*SA*^ mice (*n* = 5–6 per group). All of the data represented as mean ± SEM; ^*^P < 0.05, ^*^^*^P < 0.01. Statistical analysis: unpaired student's *t*-test **(B,G)**, non-parametric Mann-Whitney *U* test **(F,H)**.

We next performed morphometries of the atherosclerotic plaques to further understand the mechanism underlying the exacerbation of atherogenesis by the long-term RIPK1 inhibition. We found that the number of macrophages in atherosclerotic plaque was significantly increased in the long-term RIPK1i group, compared to control group (Figures 4A,B), while the number of lesional smooth muscle cells showed no difference (Figures 4C,D). No difference was observed in plaque necrotic core area (Supplementary Figures S4A,B). Plaque macrophages phagocytize oxLDL through scavenger receptors, such as CD36, transforming into foam cells, and thereby resulting in an excessive cholesterol accumulation in the intima (3). To test if RIPK1 inhibition regulates foam cell formation, peritoneal macrophages were isolated from *ApoE*^*SA*/*SA*^ and C57BL/6J mice and stimulated with oxLDL. RIPK1i markedly promoted macrophage foam cells formation *ex vivo* (Figures 4E,F). CD36 gene expression in isolated peritoneal macrophages was significantly enhanced by RIPK1i treatment (Figure 4G). Expression of lipid metabolism related genes, *Para, Lxra, Lxrb*, and *Srebp1c*, was significantly upregulated in the RIPK1i-treated macrophages, as compared to vehicle group (Figure 4H). Expression of cell-surface proteins that associate with cholesterol efflux (ABCA1, ABCG1) in macrophages did not differ between the two groups (Supplementary Figures S5A,B). These data supported that RIPK1 inhibition might promote macrophage foam cell formation via increasing lipid uptake while recruited in vessel wall/atherosclerotic plaques.

**Figure 5:**
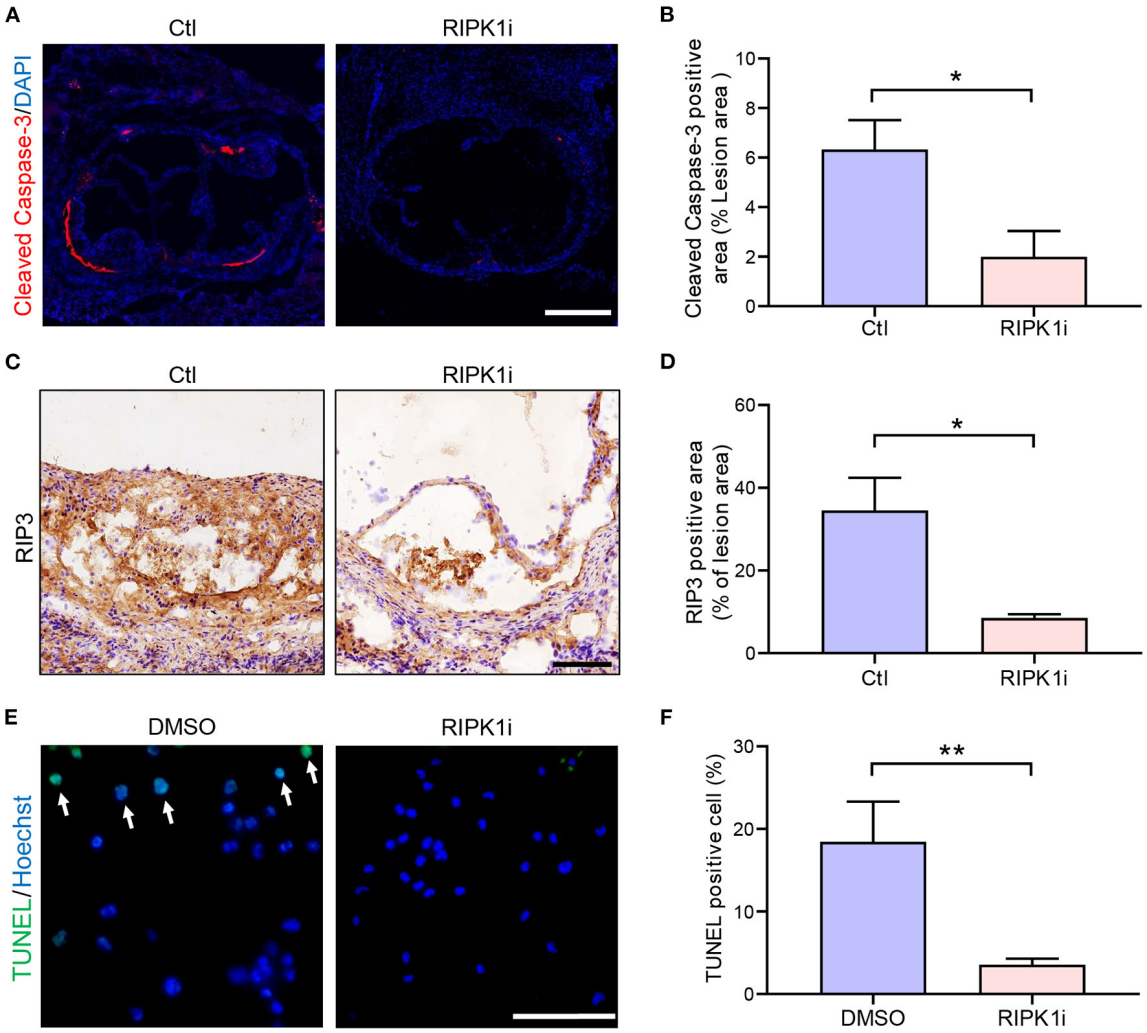
RIPK1i suppressed cell apoptosis and necroptosis in plaque. **(A–D)** Histological analysis of cross-sections from aortic sinus of *ApoE*^*SA*/*SA*^ mice. **(A)** Representative immunofluorescence images of Cleaved Caspase-3 staining (red) in aortic sinus. Scale bar = 500 μm. **(B)** Quantification of apoptosis (Cleaved Caspase-3 positive) area (*n* = 5). **(C)** Representative IHC images of RIP3 in aortic sinus plaque. Scale bar = 100 μm. **(D)** Quantification of necroptosis (RIP3 positive) area (*n* = 6). **(E)** and **(F)** Peritoneal macrophages isolated from male *ApoE*^*SA*/*SA*^ mice were pretreated with RIPK1i (50 ng/ml) or DMSO for 3 h, then stimulated with oxidized LDL (50 μg/ml) for 24 h and stained for TUNEL. **(E)** Representative immunofluorescence images of TUNEL staining (green). TUNEL positive cells are indicated by the white arrows. Scale bar = 50 μm. **(F)** Percentage of TUNEL positive cell in peritoneal macrophages (*n* = 6). All of the data represented as mean ± SEM; ^*^P < 0.05, ^*^^*^P < 0.01. Statistical analysis: unpaired student's *t*-test **(B)**, non-parametric Mann-Whitney *U* test **(D,F)**.

Inhibition of RIPK1 is known to prevent cell death (10). Here, we observed that RIPK1i significantly decreased cell apoptosis (Figures 5A,B) and necroptosis (Figures 5C,D) in cross-sections of aortic sinus plaque. Meanwhile, we also observed that the apoptosis of macrophages induced by oxLDL treatment were alleviated by RIPK1i (Figures 5E,F). Taken together, these data suggest that RIPK1 inhibition might lead to the foam cell accumulation in the plaque both by promoting foam cell formation (via upregulating lipid uptake) and by suppressing their cell death, thus exacerbating late-stage atherosclerosis.

### RIPK1i Treatment Alleviated Systemic Inflammation in Early Stage of Atherosclerosis

**Figure 6:**
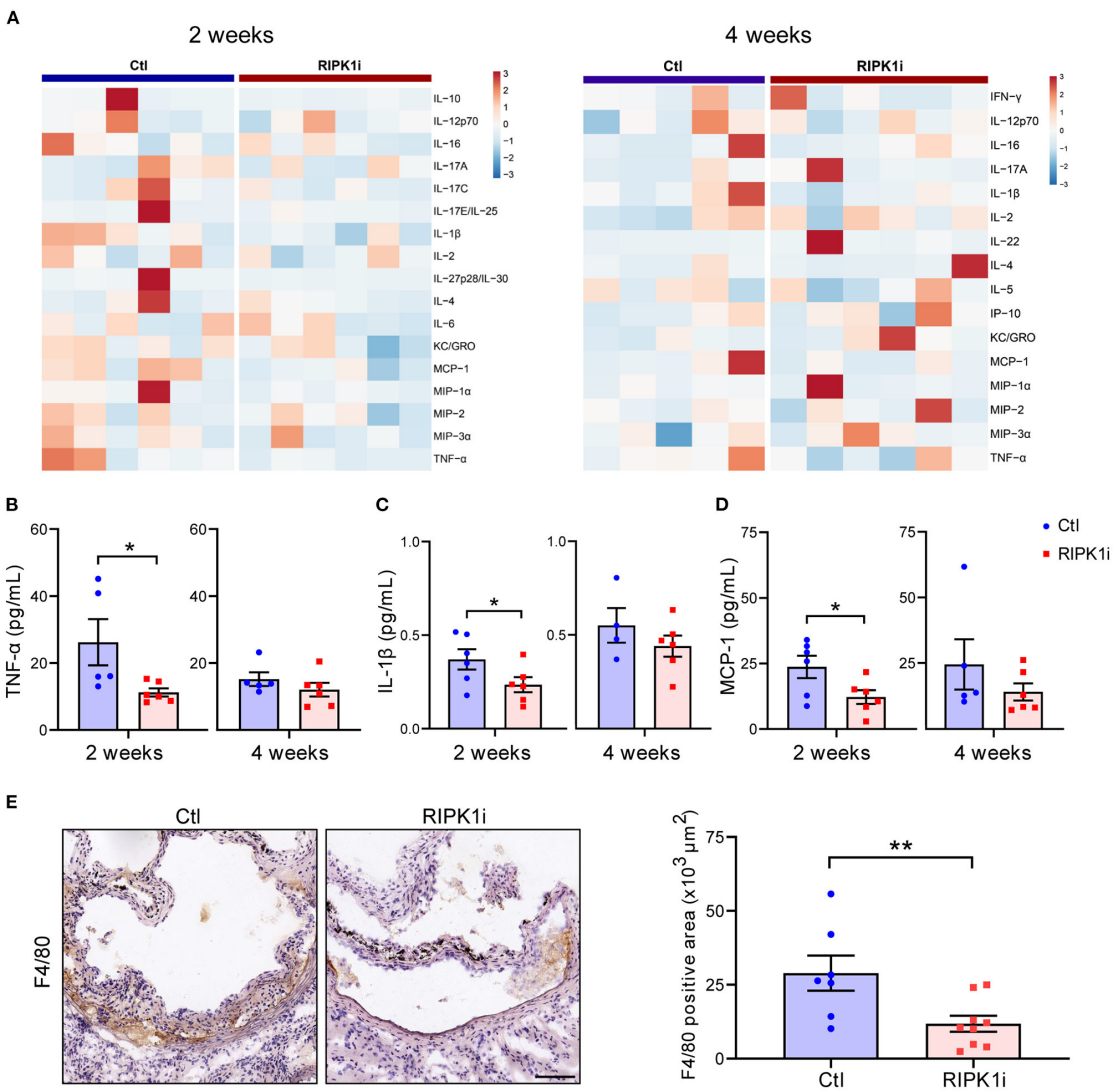
RIPK1i treatment alleviated systemic inflammation and macrophage infiltration in early atherogenesis. **(A)** Heat map analysis of inflammatory cytokines levels in *ApoE*^*SA*/*SA*^ mice in early stage (left, 2 weeks) and late stage (right, 4 weeks). **(B–D)** Plasma levels of TNF-α **(B)**, IL-1β **(C)** and MCP-1 **(D)** in early stage (2 weeks) and late stage (4 weeks) of *ApoE*^*SA*/*SA*^ mice. *n* = 4–6 mice per group. **(E)** Representative IHC images of F4/80 in aortic sinus plaque from *ApoE*^*SA*/*SA*^ mice in early stage (left). Quantification of macrophages (F4/80 positive) are represented (right). Scale bar = 100 μm. *n* = 7.9 mice per group. All of the data represented as mean ± SEM; ^*^P < 0.05; ^*^^*^P < 0.01. Statistical analysis: non-parametric Mann-Whitney *U* test **(B,E)**, unpaired student's *t*-test **(C,D)**.

Besides lipid metabolism, inflammation plays an important role in atherogenesis. To determine the influence of RIPK1i treatment on inflammatory response, we measured the plasma levels of inflammatory cytokines of mice. As shown in Figures 6A–D, plasma levels of inflammatory cytokines, including TNF-α, IL-1β, and MCP-1 levels, were substantially decreased in the RIPK1i-treated group compared to control group, in early stage atherogenesis, but not in late stage. MCP-1 was a critical chemokine for monocyte/macrophage infiltration into inflamed vessels (25). We thus examined the infiltration of macrophages in the atherosclerotic plaque of early stage by immune-staining of F4/80, and found that macrophage infiltration in aortic sinus lesions was diminished by RIPK1i treatment (Figure 6E). Thus, the early athero-protection of RIPK1 inhibition may be due to suppressed inflammation.

Lastly, to examine the effect of RIPK1 inhibition in a setting of coronary plaque rupture, which is related to clinical revascularization treatment of acute myocardial infarction, post-ischemia administration of RIPK1i reduced cardiac infarct size (Supplementary Figure S6).

## Discussion

Taking advantage of *ApoE*^*SA*/*SA*^ mice, a new model combining both hypercholesterolemia and hypertension that rapidly develops atherosclerosis (manuscript under submission), we examined the impact of RIPK1 inhibition on advanced atherosclerosis. Here, we report that RIPK1 inhibition protected against early atherosclerosis, confirming a recent observation (14), however, surprisingly, it promoted atherogenesis in late stage. This reflects a dual action of RIPK1 in inflammation and cholesterol metabolism related to foam cell formation. The early athero-protective effect of RIPK1 inhibition is consistent with reduced monocyte infiltration to atherosclerotic lesions and alleviated system inflammation, including suppressed MCP-1 production.

We uncovered a key role of RIPK1 in suppressing oxidized lipids uptake by macrophages. RIPK1 inhibition upregulated macrophage expression of *Cd36* and other lipid metabolism-related genes, including *Ppara, Lxra, Lxrb* (26), *Srebp1c* (27). Consistent with our observation, a recent study reports that MLKL (downstream of the RIPK1/RIPK3 pathway) knock-down increased lipid deposition in the plaque in *ApoE*^−/−^ mice and increased lipid accumulation in macrophages foam cells (18). Colleagues also report that RIPK1 regulates expression of CH25h, which catalyzes the rate-limiting step in the conversion of cholesterol to the oxysterol 7α, 25-dihydroxycholesterol (25HC) (28).

We found that inhibiting RIPK1 suppressed cells necroptosis and apoptosis in plaques. As atherosclerosis progressed, we found substantial increase in the number of macrophages in late stage atherogenesis plaque in RIPK1i group. This could be explained by the impact of RIPK1i in foam cell dynamics: enhanced formation of macrophages foam cells and reduced cell death. Apoptosis of macrophages plays a critical role in atherosclerosis. Genetic deletion of apoptosis inhibitor of macrophages (AIM) in *Ldlr*^−/−^ mice accelerated macrophages apoptosis in atherosclerosis lesions, which resulted in reduction of atherosclerotic plaque with decreased macrophages content (29). On the other hand, defective clearance of apoptotic debris in advanced lesions favors arterial wall inflammation and enhanced recruitment of monocytes, leading to enhanced atherogenesis (30). Thus, RIPK1i treatment may play both protective and detrimental roles in atherosclerosis depending on different stages. This is in line with previous observations in MLKL knockdown mice (18) and in Nec-1(another RIPK1 inhibitor) treated *ApoE*^−/−^ mice (17) or foam cells *in vitro* (31). Consistent with our results, myeloid-specific RIPK1 gene deletion resulted in differential impacts at different atherogenesis stages (15).

This study also reveals a key role of RIPK1 in liver cholesterol metabolism. HDL cholesterol level was reduced consistently under RIPK1 administration. Accordingly, liver expression of ApoA1, the dominant structural protein component of HDL (32), was also suppressed by RIPK1i. In addition, the expression level of hepatic PPARα was inhibited, which might explain the decreased ApoA1 synthesis (7). HDL transports excess lipid from macrophage/foam cells in atherosclerotic lesions to liver (6), attenuating atherosclerosis progression (33). Therefore, RIPK1i might reduce HDL via inhibition of liver synthesis of ApoA1, contributing to the pathogenesis of atherosclerosis at a later stage.

Although we provided evidence that RIPK1 regulated the synthesis of ApoA1 in the liver possibly through PPARα, the exact underlying mechanism warrants future investigation. Also, unclear in this study is whether RIPK1i might prevent the endothelial dysfunction or cell death, contributing to the retarded atherogenesis in early stage.

**Figure 7:**
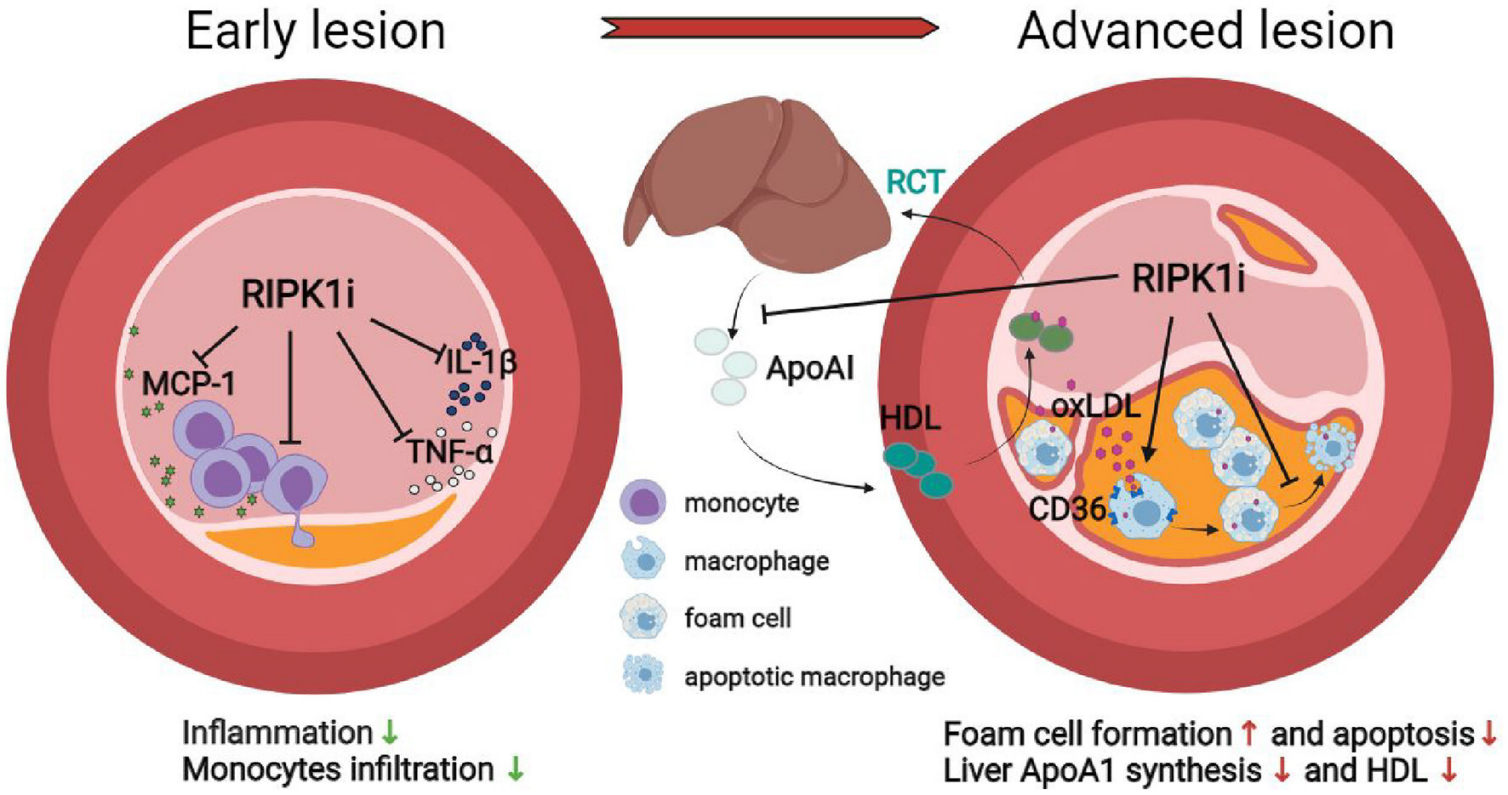
Schematic illustration of the stage-dependent impact of RIPK1i in atherosclerosis.

In summary, we report a stage-dependent effect of RIPK1 inhibition in atherogenesis under two most common risk factors—hypercholesterolemia and hypertension. Short-term RIPK1 inhibition diminished macrophage infiltration and inflammatory response, preventing early stage atherogenesis, whereas extended drug exposure increased foam cell formation, reduced HDL cholesterol, resulting in lipid accumulation in lesion area, and exacerbated late atherogenesis. RIPK1i administration may play both protective and detrimental roles in atherosclerosis depending on different stages. The stage-dependent impact of RIPK1 inhibition on atherogenesis and related mechanisms are schematically illustrated (Figure 7). These results call for attention to define optimal therapeutic timing/duration for future clinical use of RIPK1 inhibitors in cardiovascular diseases, as well as in treating other RIPK1-driven diseases with cardiovascular concern.

## Data Availability Statement

The original contributions presented in the study are included in the article/Supplementary Material, further inquiries can be directed to the corresponding author/s.

## Ethics Statement

The animal study was reviewed and approved by Institutional Animal Care and Use Committee, the Experimental Animal Center, Fuwai Hospital, National Center for Cardiovascular Diseases, China.

## Author Contributions

YZ designed and performed most of the experiments and wrote the manuscript. HL contributed to peritoneal macrophages isolation and histological analysis. HC designed the primers and wrote the methods of lipid measurements. HR, YH, GY, QW, and ZP assisted in animal study, sample collection, and analysis. JB, BG, MR, and JT contributed to pharmacokinetics of RIPK1 inhibitor and critically revised the manuscript. MW conceived and supervised the study and critically revised the manuscript.

## Funding

This work was supported by the National Key Research and Development Program of China (2020YFC2008000), Chinese Academy of Medical Sciences Innovation Fund for Medical Sciences (2021, 2017-I2M-1-008, 2016-I2M-1-003/006), and research funds from Fuwai Hospital, Peking Union Medical College and Chinese Academy of Medical Sciences.

## Conflict of Interest

JB, BG, MR, and JT are current or previous employee at GSK. The remaining authors declare that the research was conducted in the absence of any commercial or financial relationships that could be construed as a potential conflict of interest.

## Publisher's Note

All claims expressed in this article are solely those of the authors and do not necessarily represent those of their affiliated organizations, or those of the publisher, the editors and the reviewers. Any product that may be evaluated in this article, or claim that may be made by its manufacturer, is not guaranteed or endorsed by the publisher.
