# Supplementary material for: Stage-Dependent Impact of RIPK1 Inhibition on Atherogenesis: Dual Effects on Inflammation and Foam Cell Dynamics
Source: Front Cardiovasc Med. 2021 Oct 25;8:715337. doi: 10.3389/fcvm.2021.715337 (PMC8572953; doi:10.3389/fcvm.2021.715337)
Supplement: Supplementary file 1 [file Data_Sheet_1.PDF]

Supplementary materials

Stage-dependent impact of RIPK1 inhibition on atherogenesis: dual effects on inflammation and foam cell dynamics

Zhang et al.

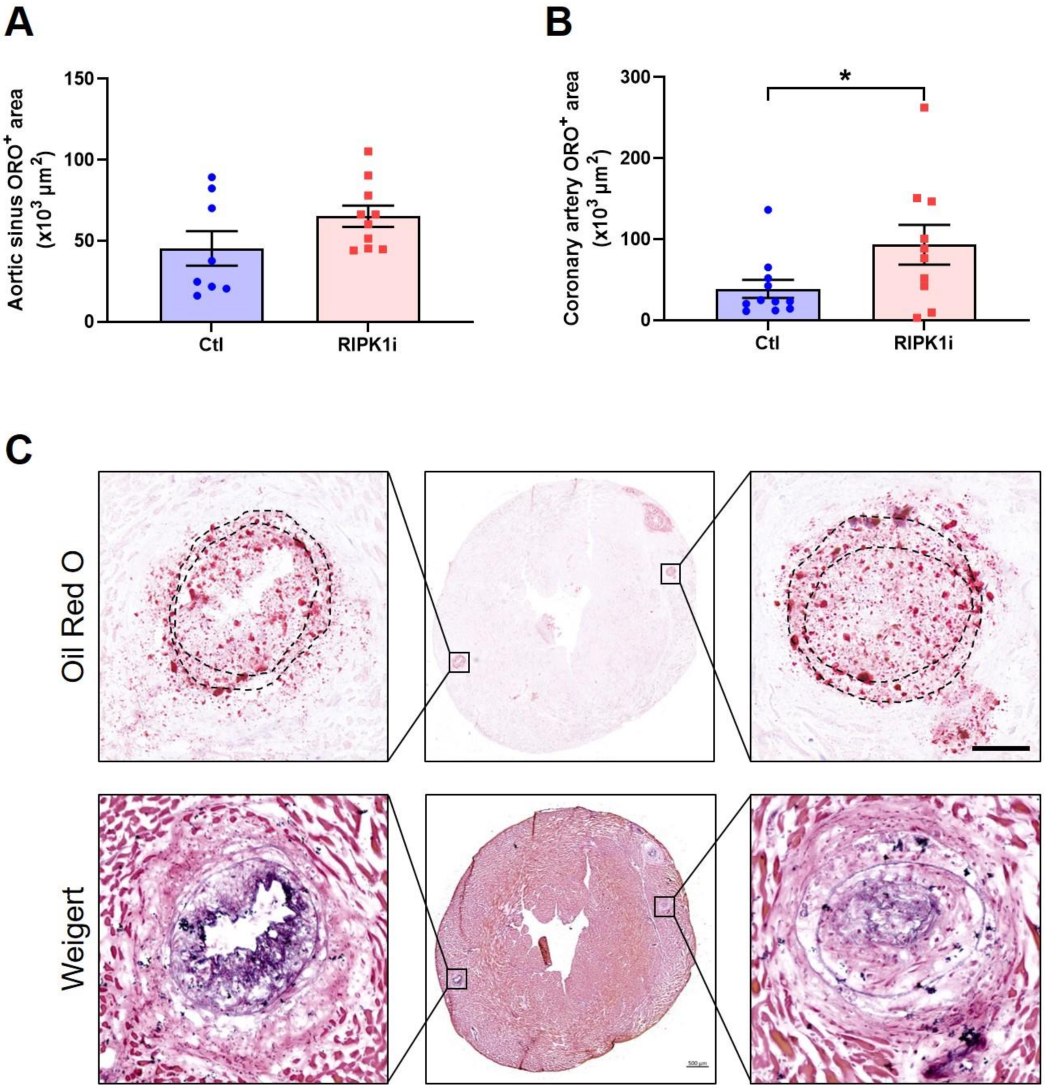

Figure S1. RIPK1i increased lipids deposition in late stage of atherosclerosis.
Quantification of aortic sinus (**A**) and coronary artery (**B**) ORO-positive area of mice in late stage (4 weeks). n=8-11 female mice per group. All the data represented as mean ± SEM; *P<0.05. Statistical analysis: nonparametric Mann-Whitney U test. Panel C: Oil Red O staining (top) and Weigert staining (bottom) of coronary arteries in male *ApoE*$^{SA/SA}$ mice treated with RIPK1i for 4 weeks. Dashed lines showed vessel media. Scale bar=50μm.

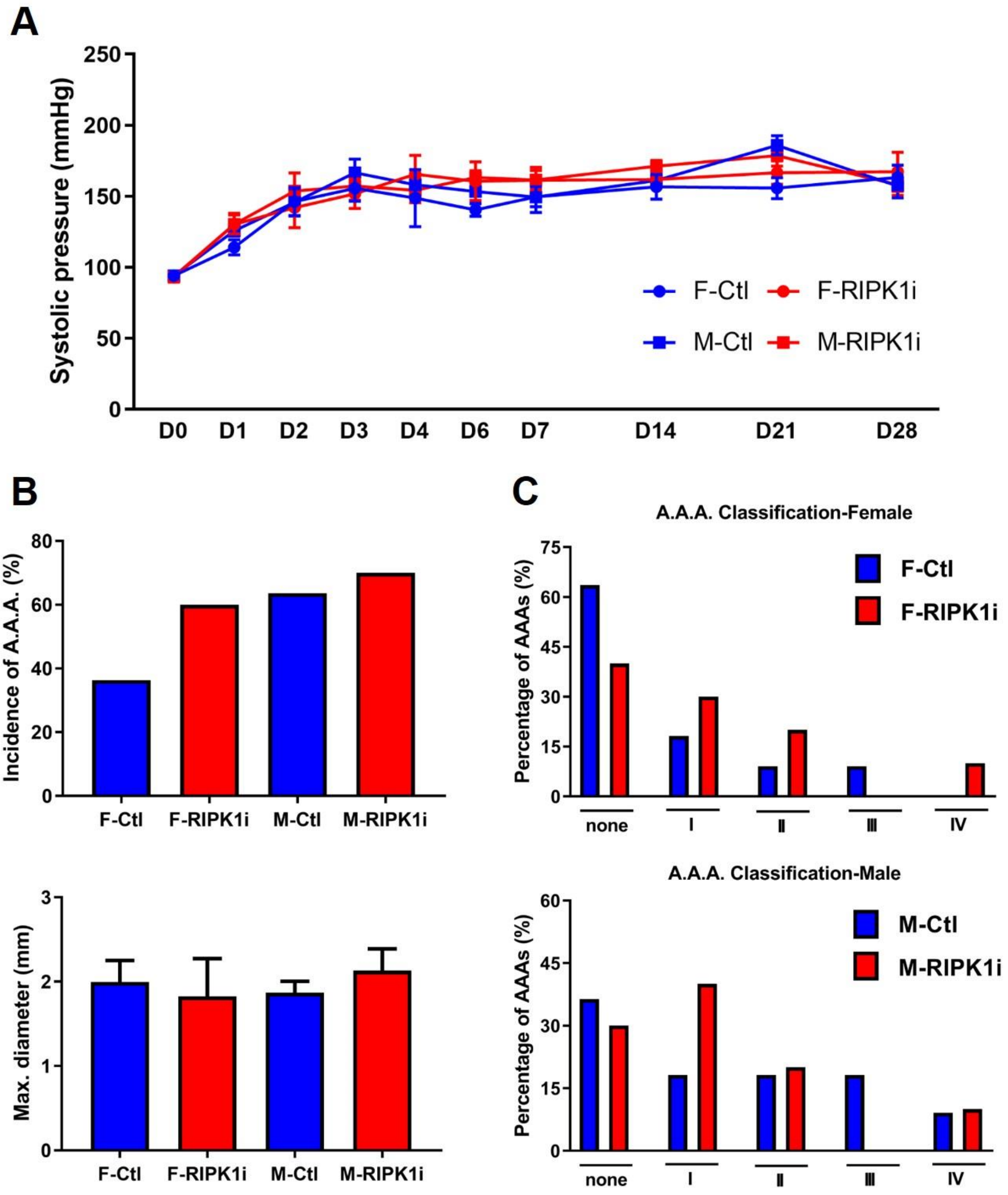

Figure S2. RIPK1i did not affect hypertension and abdominal aortic aneurysm (AAA) formation.

A) Systolic blood pressures of female (n=11) and male (n=15) *ApoE*$^{SA/SA}$ mice treated with 4 weeks. **B)** and **C)** Abdominal aortic aneurysm analysis of female (n=11) and male (n=15) *ApoE*$^{SA/SA}$ mice treated with 4 weeks. **B)** Incidence (up) and maximal diameter (bottom) of abdominal aortic aneurysm. **C)** Percentage of different types of abdominal aortic aneurysms. F=female, M=male.

,

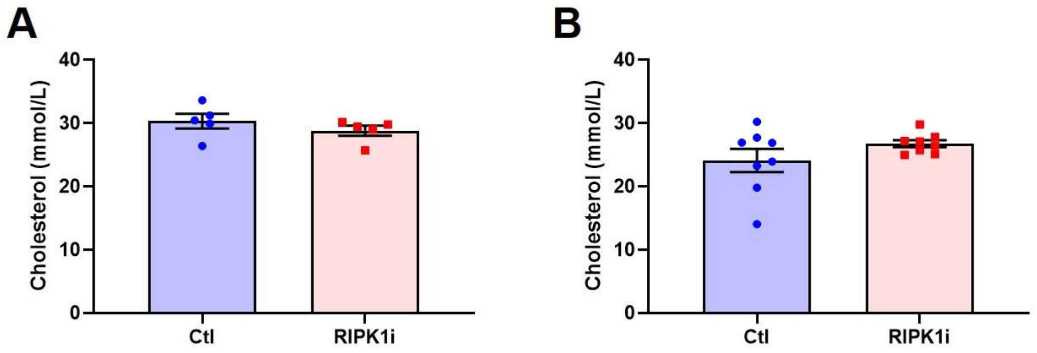

Figure S3. RIPK1i treatment did not change total cholesterol level.
Plasma total cholesterol level in female (**A**) and male (**B**) *ApoE*$^{SA/SA}$ mice during atherogenesis. n=5-8 mice per group.

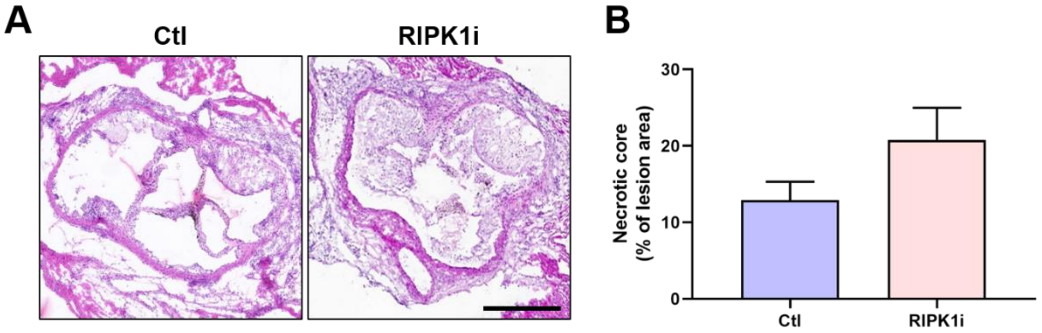

Figure S4. RIPK1i treatment did not change plaque vulnerability
Representative histological analysis of cross-sections from the aortic sinus stained with hematoxylin and eosin (H&E) (**A**, scale bar=500μm). Quantification of the necrotic core (**B**).

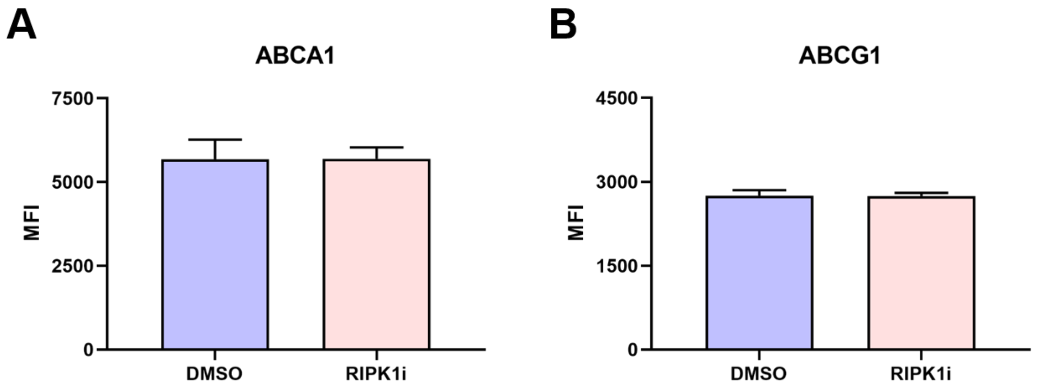

Figure S5. Expression of ABCA1 and ABCG1.
Detection of cell surface ABCA1 (**A**) and ABCG1 (**B**) expression by FACS (n=5).

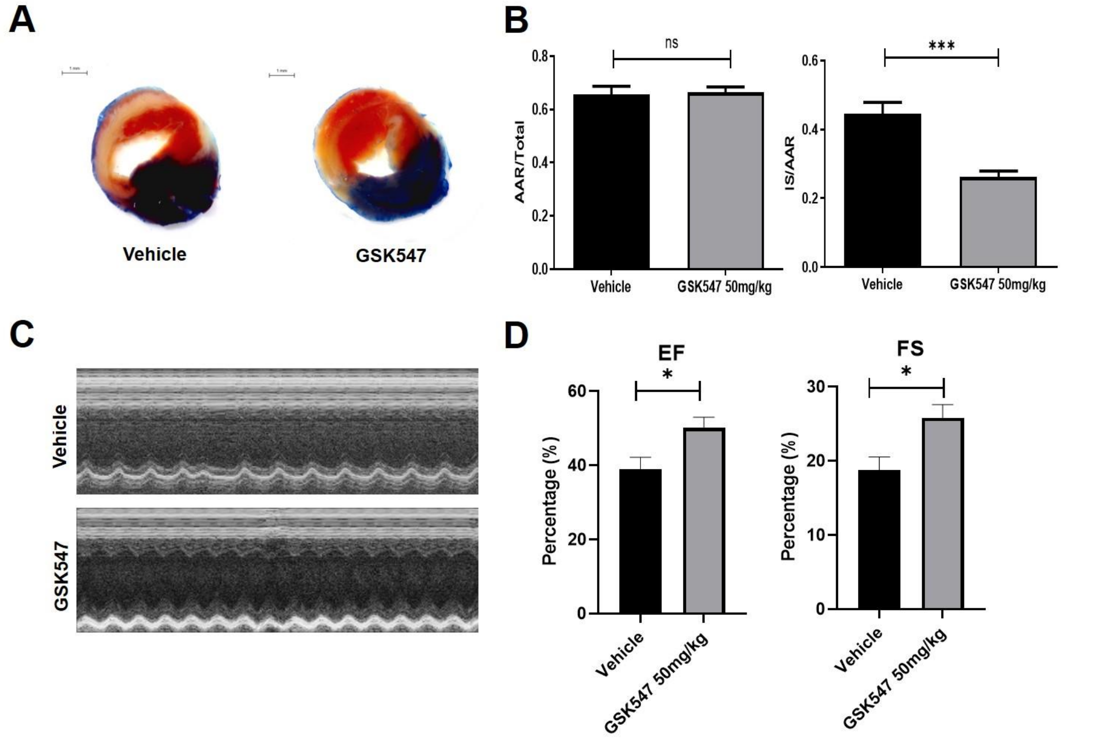

Figure S6. The effect of RIPK1 inhibition in myocardial ischemia reperfusion.

Representative photos of TTC stained Evans blue perfused hearts were shown (**A**). AAR/Total ratio (**B**, left) and infarct size/AAR ratio (**B**, right). **C)** Representative M-Mode echocardiography of mice. **D)** Ejection fraction(left) and fraction shortening(right). n=11-12 mice per group. All of the data represented as mean ± SEM; *P<0.05, ***P<0.001. Statistical analysis: unpaired student's t test.
